# Probable invasive *Alternaria* rhinosinusitis in a previously healthy patient diagnosed with heterozygous CARD9 deficiency and combined immunodeficiency

**DOI:** 10.1016/j.idcr.2026.e02763

**Published:** 2026-09-16

**Authors:** Chelsea Morinishi, Omai B. Garner, Shangxin Yang, Paul R. Allyn

**Affiliations:** a Division of Infectious Diseases, Department of Internal Medicine, David Geffen School of Medicine at UCLA, 924 Westwood Blvd., #200, Los Angeles, California, 90024, United States; b Department of Pathology and Laboratory Medicine, David Geffen School of Medicine at UCLA, 11633 San Vicente Blvd, Los Angeles, CA, 90049, United States

**Keywords:** Invasive fungal rhinosinusitis, Molecular diagnostics, Immunocompromised, Dematiaceous mold, Alternaria, CARD9 mutation

## Abstract

*Alternaria* is a dematiaceous mold emerging as a cause of invasive fungal infections in immunocompromised patients including invasive fungal rhinosinusitis. Prior case reports have described severe fungal infections in seemingly healthy patients later diagnosed with autosomal recessive CARD9 (caspase recruitment domain-containing protein 9) mutations. Here we report a case of invasive rhinosinusitis due to dematiaceous mold in a previously healthy patient with heterozygous CARD9 mutation and combined immunodeficiency cured with surgical resection and triazole therapy. The causative pathogen was most likely *Alternaria* species based on histopathologic findings and Next-Generation Sequencing results despite negative tissue cultures. We performed a retrospective review of all adult patients at our institution with *Alternaria* species identified by culture or polymerase chain reaction and found no other instances of invasive fungal rhinosinusitis. While highlighting the diagnostic dilemmas of our case, we present a comprehensive review of the available literature regarding management and clinical outcomes of dematiaceous mold infections, particularly invasive *Alternaria* rhinosinusitis, and the clinical implications of CARD9 deficiency.

## Highlights

• Dematiaceous molds are a cause of invasive rhinosinusitis in the immunocompromised.
• Molecular diagnostics are sensitive but can introduce diagnostic uncertainty.
• Surgical debridements and adjunctive triazole therapy led to clinical cure.
• In healthy patients, immunologic evaluation can reveal primary immunodeficiencies.

## Case report

A 51-year-old woman with a history of chronic allergic rhinitis, nasal polyposis, and frequent childhood ear infections presented to our hospital with five months of worsening nasal pain and crusting. Prior to onset, she had an extensively documented, recent history of recurrent maxillary and ethmoid sinusitis for which she was treated by otolaryngology with short courses of oral antimicrobials and steroids. Due to a lack of clinical response, she underwent bilateral middle turbinectomy, uncinectomy, and polypectomy three years before presentation. Tissue histopathology at this time had demonstrated chronic sinusitis, sinonasal inflammatory polyps, and granulomatous inflammation with numerous fungal organisms highlighted by Grocott’s methenamine stain (GMS), but no antifungal treatment was initiated due to negative fungal tissue cultures. During the following three years, she developed progressively worsening nasal congestion refractory to a combination of saline rinses, fluticasone and azelastine nasal sprays, and courses of oral steroids plus antimicrobials. Five months prior to presentation, she noticed increased nasal crusting, purulent nasal drainage, and gum swelling. She presented to an outside hospital one month prior to admission and underwent biopsy of the right maxillary sinus that demonstrated chronic granulomatous inflammation with Periodic Acid-Schiff (PAS) and GMS staining with septate, filamentous microorganisms. There was concern for possible granulomatosis with polyangiitis, for which she was treated with another course of prednisone without antifungal therapy by the outside treating physicians. Within one month, she developed progressive disease with worsening sinus pain, intermittent epistaxis, anosmia, and a new oronasal fistula, prompting presentation to our hospital for expedited multidisciplinary care.

On arrival, physical examination was remarkable for marked gingival recession, an open hard palate defect with surrounding erythema, and bilateral crusting of the nares (Fig. 1). Nasal endoscopy revealed moderate nasal crusting in the right nasal cavity greater than the left, but no areas of necrosis. Laboratory findings showed elevated C-reactive protein of 1.5 mg/dL and a positive ANA of 1:320 but were otherwise unremarkable. Computed tomography (CT) of the face (Fig. 2) showed extensive left greater than right opacification of the maxillary, ethmoid, and frontal sinuses with oronasal fistula and erosive changes of the bilateral medial orbital walls and hard palate. *Citrobacter freundii* and *Pseudomonas aeruginosa* grew on superficial sinus cultures. The Infectious Diseases team started isavuconazole (ISAV) given its more favorable side effect profile and broad fungal activity based on the outside pathology report noted above. Cefepime was also initiated for targeted bacterial coverage.

**Fig. 1:**
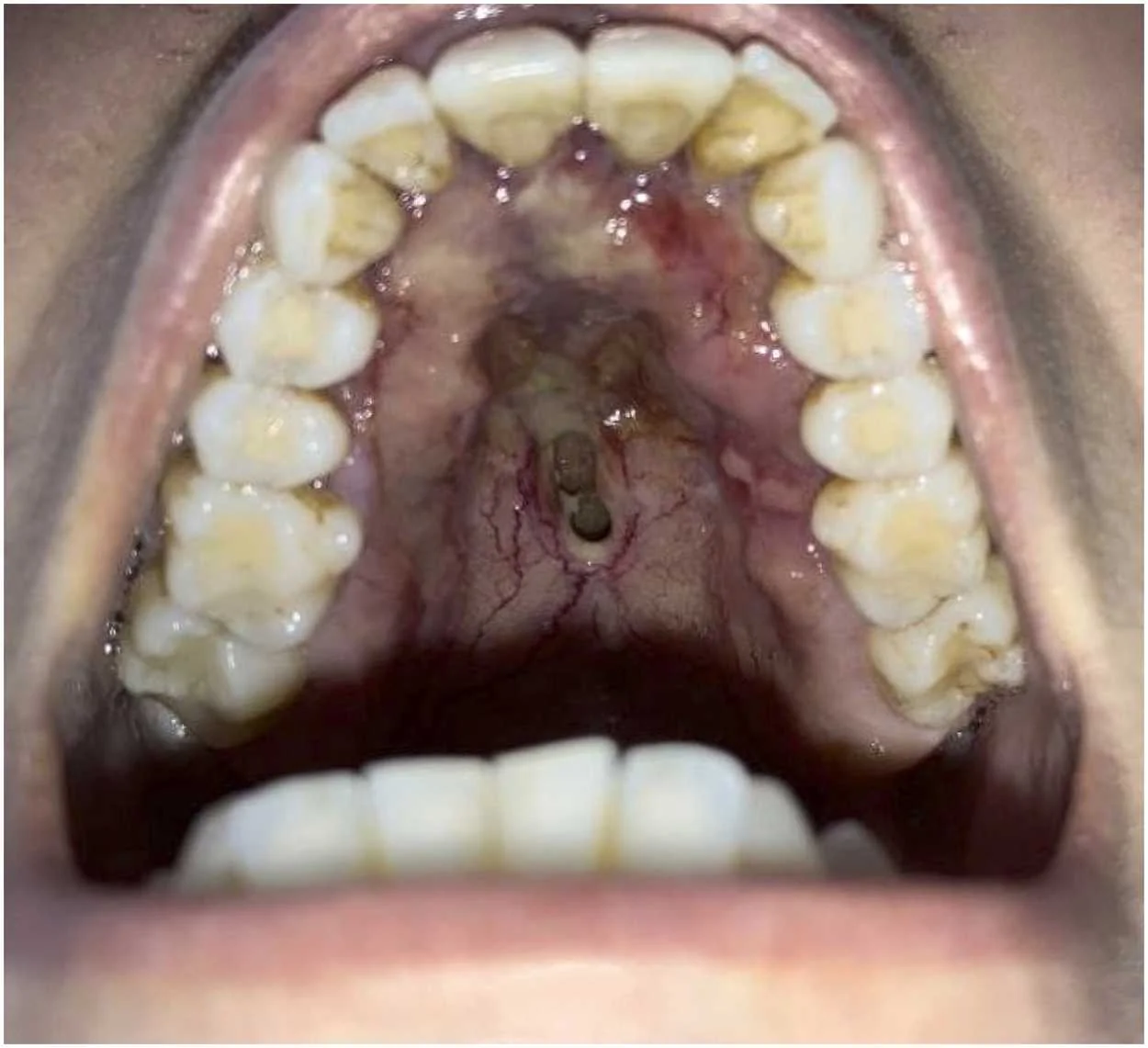
Oronasal fistula with surrounding hyperemia.

**Fig. 2:**
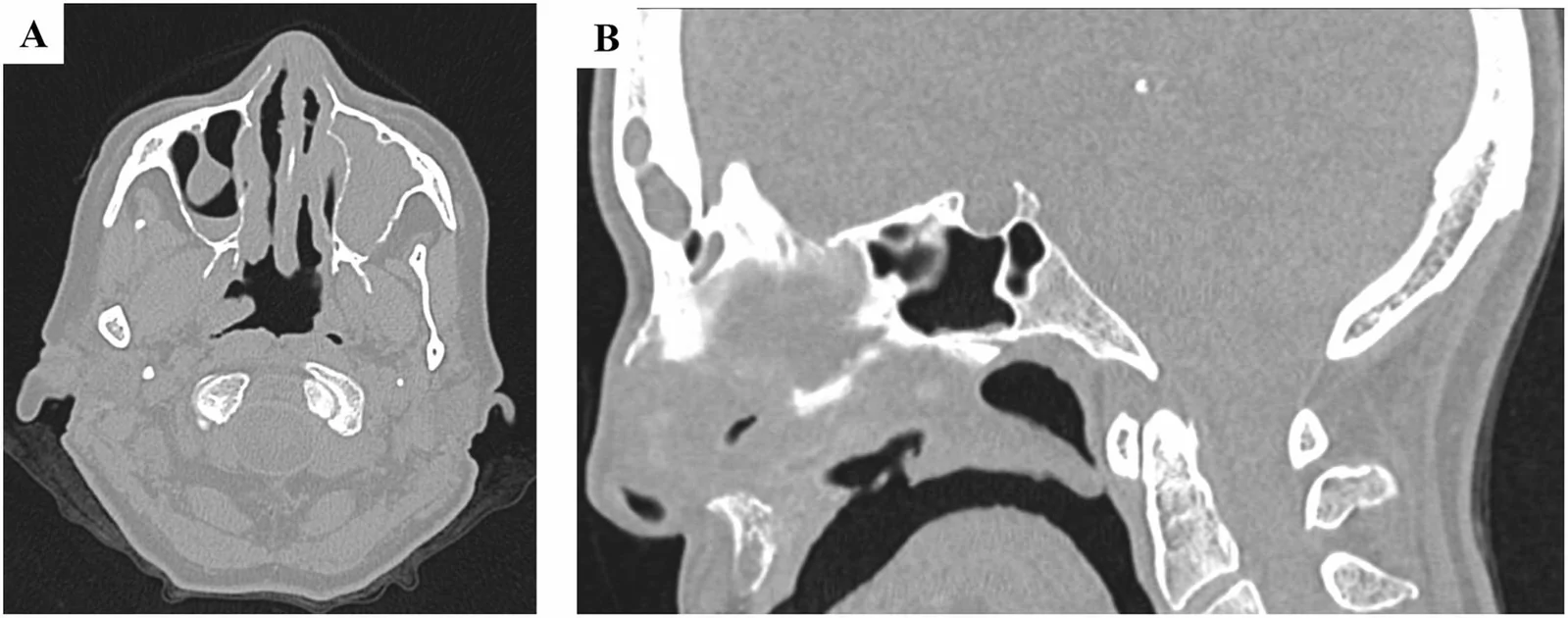
Axial and sagittal views of the CT showing (A) complete opacification of the left maxillary sinus and (B) erosion of the hard palate with fistulization along the posterior hard and soft palate.

Sinus debridement was performed on hospital day 10, wherein septal perforation and extensive necrotic tissue were seen in the maxillary and ethmoid sinuses (Fig. 3). Tissue pathology (Fig. 4A-B) with GMS staining showed septate, acute angle branching fungal organisms with globose structures, and tissue invasion. Due to the severity of her disease and the increasing concern for invasive fungal sinusitis including Mucormycosis, liposomal amphotericin B (L-AmB) was added to her antifungal regimen. Broad tissue cultures were obtained and additional specimens were sent to the University of Washington for AFB, bacterial, and fungal PCR with reflex to Next-Generation Sequencing (NGS). Surgical fungal and AFB cultures ultimately did not grow. On further review of the pathology slides, globose swelling was noted along the hyphae, which increased suspicion for a dematiaceous mold. After the addition of the Fontana Masson stain to differentiate dematiaceous molds from hyaline molds, the fungal organisms stained positive (Fig. 4*C*).

**Fig. 3:**
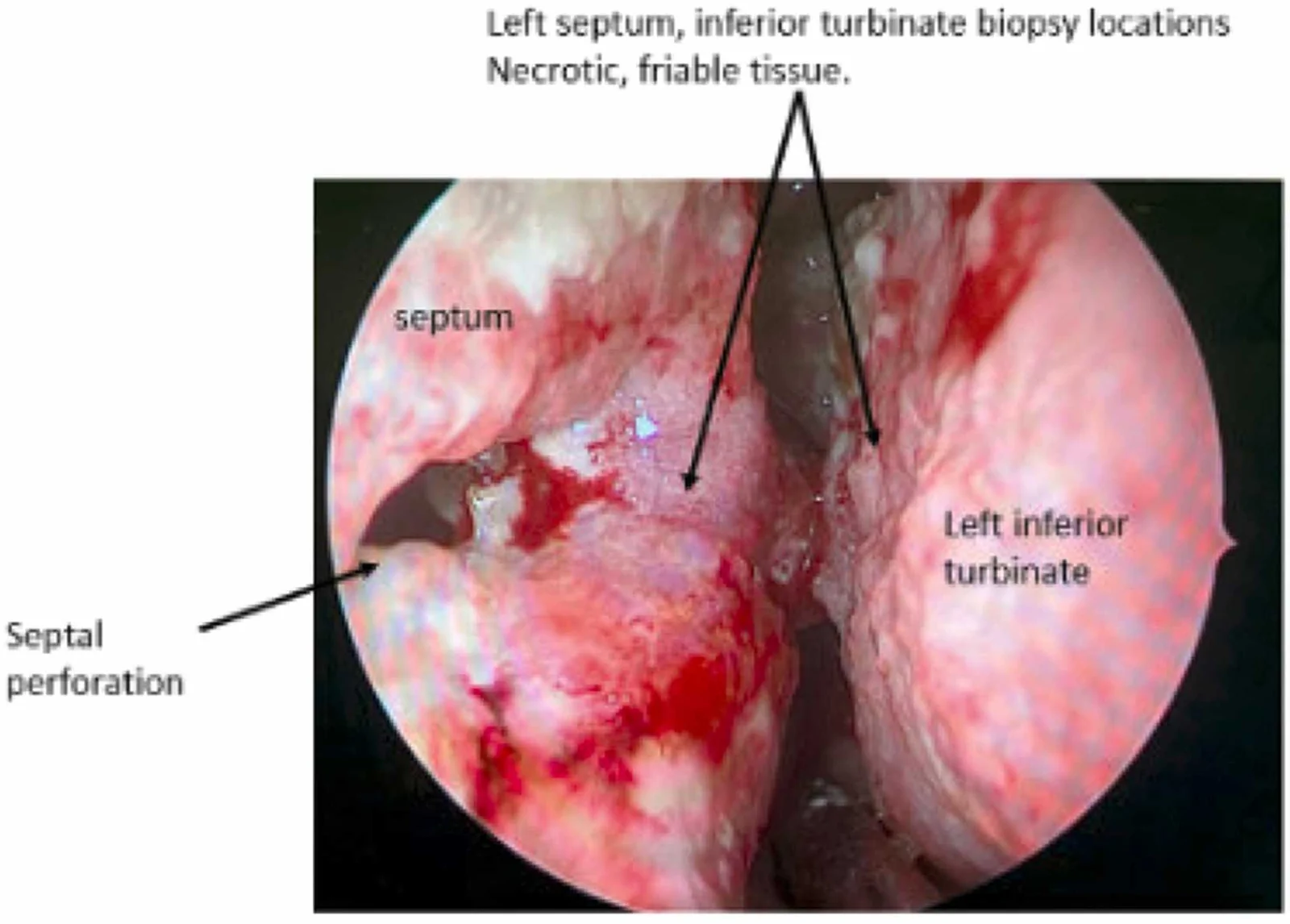
Operative view of septal perforation and necrotic tissue of left inferior turbinate.

**Fig. 4:**
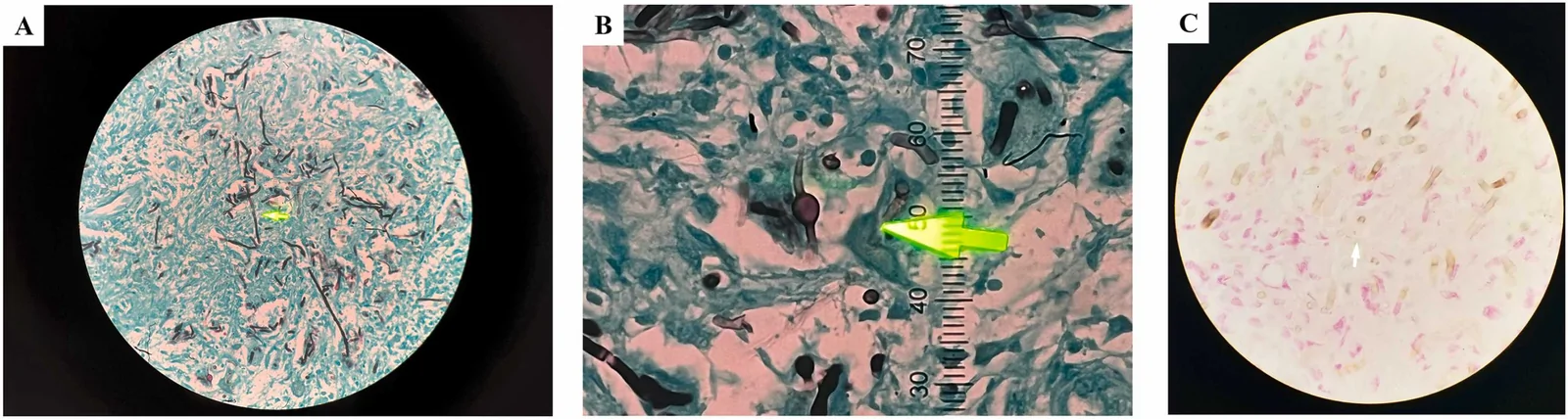
Pigmented, septate hyphae staining positive with Grocott’s methenamine stain (A-B) and Fontana-Masson stain (C).

L-AmB was discontinued seven days after the first debridement based on the above pathology results. The patient was discharged with ISAV 372 mg daily and 14 days of ciprofloxacin. A few weeks after discharge, tissue fungal NGS results showed *Alternaria* spp., *Candida krusei*, *Botryotrichum* species, *Malassezia restricta*, and *Pichia barkeri*. *Candida krusei, Malassezia restricta, and Pichia barkeri* were suspected to be either colonizers or contaminants due to the absence of yeast on tissue pathology. *Alternaria* spp. was determined to be the most probable causative pathogen rather than *Botryotrichum* species as the histopathologic findings (Fontana Masson positive and globose structures) were not consistent with *Botryotrichum*. She underwent four additional debridements in the outpatient setting given the extent of her disease. After 40 days, ISAV was switched to posaconazole (POS) 300 mg daily due to peripheral edema, which was attributed to the drug. Prior data also showed a more favorable minimum inhibitory concentration (MIC) for POS compared with ISAV. As she demonstrated radiographic and endoscopic improvement, posaconazole was discontinued at 10.5 months from initial debridement. She remained disease-free at 17 months and successfully underwent reconstructive palate surgery.

Early in her outpatient course, the patient was concurrently evaluated by Immunology for possible primary immunodeficiency disorder. Initial blood tests showed low absolute lymphocyte count (0.96 ×10 ³/µL). Subsequent workup included lymphocyte enumeration and immunoglobulin levels which revealed lymphopenia (CD3 636, CD4 420, and CD8 185) and panhypogammaglobulinemia (IgG 662, IgM 34, and IgA 73). Considering the increased risk for bacterial and fungal infections, Mendelian susceptibility to mycobacterial disease, chronic granulomatous disease, leukocyte adhesion deficiency, and cystic fibrosis were also considered, but workup was negative. Whole exome sequencing was then performed for genetic analysis of her combined immunodeficiency, which revealed a heterozygous variant in the CARD9 (caspase recruitment domain-containing protein 9) gene (c.723dupG). Mitogen/antigen proliferation testing revealed low T-cell response to Candida and tetanus antigens, indicating T-cell dysfunction. She initiated subcutaneous immunoglobulin injections every two weeks with normalization of IgG and IgA levels, but persistently low IgM. Her immunizations were updated accordingly, including Pneumococcus, RSV, COVID-19 and influenza.

## Methods

With approval from the Institutional Review Board, a retrospective chart review was performed of all adult patients with positive sinus cultures, tissue PCR, and/or blood microbial cell-free DNA for *Alternaria* spp. at our institution since 2004. Invasive rhinosinusitis was defined as invasion of vasculature or extra-sinonasal involvement of tissues and bone based on pathology, imaging, and/or endoscopic visualization. Clinical characteristics were assessed including age, comorbidities (such as diabetes, hematologic malignancy, neutropenia, solid organ or stem cell transplant), radiographic and endoscopic evaluations, surgical management, antifungal selection and duration. Cure, the primary clinical outcome, was defined as pathology confirming clean surgical margins and/or resolution of symptoms. Recurrence was also assessed through review of clinical notes, imaging, and endoscopy reports. Patients with polymicrobial growth on sinus cultures and positive sinus cultures without radiographic or clinical evidence of invasive disease were excluded.

In addition, a literature review was performed to identify additional adult cases of invasive *Alternaria* rhinosinusitis and to evaluate for specific risk factors and treatment outcomes related to medical and surgical interventions. A search of Google Scholar and PubMed was performed with the following keywords: “Alternaria”, “invasive rhinosinusitis”, “antifungal”, “immunocompetent,” “immunocompromised,” and “treatment outcomes.” The search yielded case reports and series in English dating from 1981 to 2026. Case studies with pediatric patients, chronic allergic fungal sinusitis, non-invasive rhinosinusitis, incomplete treatment information, and non-English language were excluded.

## Results

From our institution, we identified 27 sinus cultures positive for *Alternaria* species in adult patients. There were no qualifying cases that utilized blood microbial cell-free DNA for diagnosis. On chart review of the patients’ clinical history, imaging, and pathology results, no additional cases of invasive fungal rhinosinusitis were identified other than the case described.

Our literature review yielded 19 case reports and 1 case series of invasive *Alternaria* rhinosinusitis published between 1977 and 2026 (Table 1). A majority were young patients with a median age of 36 (23 – 74). Among 24 individual cases, a majority were immunocompromised (16/24 cases, 67%) with neutropenia as a frequent concurrent risk factor (13/16 cases, 81%). The degree of neutropenia was not consistently reported. Hematologic malignancy or disorders comprised 88% (14/16 cases) and stem cell transplant (SCT) recipients comprised 44% (7/16 cases) of immunocompromised patients [[1], [2], [3], [4], [5], [6], [7], [8]]. Only one patient was diagnosed with a primary immunodeficiency (homozygous CARD9 deficiency) [9]. In contrast, a minority of cases (8/24 cases, 33%) lacked any identifiable risk factors for invasive fungal infection [[10], [11], [12], [13], [14], [15], [16]]. *Alternaria* spp. were identified by routine culture in all cases and tissue invasive disease was confirmed by imaging and/or histopathology. Clinical manifestations varied from a necrotic septal lesion to oronasal fistula.

**Table 1:** Prior reports of invasive *Alternaria* rhinosinusitis.

**Author/Year**	**Study**	**Age/ Sex**	**Risk Factors**	**Diagnosis**	**Extent of Disease**	**Surgery (n)**	**Antifungal Therapy (days)**	**Outcome**
**Present Case**	Case Report	51 F	Heterozygous CARD9 mutation, defective T-cell function, hypogammaglobulinemia	Fungal NGS of sinus cultures, imaging, histopathology	Invasion of maxillary sinuses, nasal septum perforation and oronasal fistula	Yes (5)	L-AmB 5 mg/kg/day (6), then ISZ 372 mg qday (44), then POS 300 mg qday (290)	Cure, no disease at 17 months
**Sikora et al. 2024** [17]	Case Report	35 M	AML, SCT, neutropenia	Sinus cultures, PCR, histopathology	Invasion of bilateral middle turbinates	Yes (1)	L-AmB 5 mg/kg/day (18) and POS 300 mg qday, then POS (27) + micafungin (14)	Death due to AML and other infectious complications
**Pereira et al. 2022** [18]	Case Report	74 M	Aplastic anemia, neutropenia	Sinus cultures, histopathology	Invasion of right middle turbinate and posterior maxillary sinus	Yes (1)	AmB 5 mg/kg/day + POS 300 mg qday (unknown), then prophylactic POS (lifelong)	Cure, no follow up
**Tyrellis et al. 2022** [8]	Case Report	36 F	ALL, neutropenia	Sinus cultures, PCR, histopathology	Invasion and necrosis of maxillary and ethmoid sinuses	Yes (1)	L-AmB 5 mg/kg/day (38)	Cure, no disease at 6 months
**Paccoud et al. 2022** [9]	Case Report	28 F	Homozygous CARD9 mutation	Sinus cultures, imaging, histopathology	Left maxillary sinusitis with hard palate necrosis and oronasal fistula	Yes (2)	L-AmB (42), then L-Amb (91) after relapse, then L-AmB 5 mg/kg/qday (unknown), then ITR 400 mg qday (365)	One relapse, then cure, no disease at 8 years
**Hattab et al. 2019** [10]	Case Report	19 M	None	Sinus cultures, imaging	Invasion of maxillary and frontal sinuses and orbits	Yes (1)	AmB 37 mg/kg/day (15) then ITR 200 mg qday (60)	Cure, no disease at 1 year
**Chhabra et al. 2013** [19]	Case Report	55 F	DM2	Sinus cultures, imaging, histopathology	Maxillary sinusitis, osteomyelitis and oronasal fistula	Yes (1)*	Fluconazole 150 mg qday (28), then AmB (10)	Cure, no follow up
**Aruch et al. 2012** [7]	Case Report	48 M	APML, neutropenia	Sinus culture, histopathology	Necrotic lesion on hard palate and rhinosinusitis	Yes (1)	AmB unknown dose (unknown) + VOR unknown dose (indefinite)	Cure, no follow up
**Robert et al. 2012** [11]	Case Report	41 F	None	Sinus cultures, fungal PCR, histopathology	Hard palate ulceration	No	ITR 400 mg qday (365), then POS 400 mg BID after relapse (unknown)	Relapse at 5 months
**Mullane et al. 2007** [6]	Case Report	33 M	AML, neutropenia	Sinus cultures, imaging	Maxillary sinusitis and right lung infiltrates	Yes (1)	VOR 200 mg BID (21), then L-AmB 450 mg qday (42), then POS unknown dose (152), then prophylactic POS 400 mg BID (547)	Cure, no disease at 18 months
**Chen et al. 2004** [5]	Case Report	56 F	AML, neutropenia	Septal cultures, histopathology	Necrotic lesion on anterior septum	Yes (1)	AmB (unknown)	Cure, death due to unrelated causes at 3 months
**Iwen et al. 1997** [4]	Case Report	35 F	AML, neutropenia	Nasal cultures, histopathology	Nasal necrosis	Yes (1)	AmB 1 g with KET (unknown)	Cure, no follow up
**Morrison and Weisdorf 1993** [3]	Case Series	38 M	CML, SCT, neutropenia	Cultures (either blood or tracheobronchial tree), histopathology	Necrotic lesion on nasal septum	Yes (1)	AmB 1 mg/kg/day (unknown)	Cure, death due to unrelated causes
		25 M	ALL, SCT, neutropenia	Sinus cultures, histopathology	Ulcer on nasal septum	Yes (1)	AmB 1 mg/kg/day + 5-FU (unknown)	Cure, no follow up
		26 F	AML, SCT, neutropenia	Sinus cultures, histopathology	Necrotic lesion on middle turbinate	Yes (1)	AmB 1 mg/kg/day (unknown)	Cure, no follow up
		37 F	Aplastic anemia, SCT, neutropenia	Sinus cultures, histopathology	Necrotic lesion on nasal septum	Yes (1)	AmB 1 mg/kg/day (unknown)	Death due to post-operative complications
		39 F	SCT, GVHD	Sinus cultures, histopathology	Nasal crusting	Yes (1)	AmB 1 mg/kg/day (unknown)	Cure, no follow up
**Zieske et al. 1991** [12]	Case Report	21 M	None	Sinus cultures, histopathology	Chronic pansinusitis	Yes (2)	AmB 1 mg/kg/day (unknown)	Cure, no disease at 5 months
**Bartell et al. 1988** [2]	Case Report	33 M	APL, SCT, neutropenia	Cultures (nasal aspirate, skin, tissue)	Maxillary sinusitis, necrotic nasal ulcer	No	AmB unknown dose (19)	Cure, no follow up
**Goodpasture et al. 1987** [13]	Case Report	31 M	None	Sinus culture, histopathology	Hard palate ulcer and osteomyelitis, maxillary sinusitis	Yes	Miconazole unknown dose (26), then AmB 50 mg TIW (60) after relapse	One relapse, then cure at 2 years
**Murtagh et al. 1987** [14]	Case Report	51 M	None	Culture (orbital mass), imaging, histopathology	Left orbital swelling, osteomyelitis of orbital bone and maxillary sinusitis	Yes (2)	AmB unknown dose (42), AmB unknown dose (56) after first relapse, then AmB unknown dose (730) after second relapse	Two relapses, then cure at 3 years
**Wiest et al. 1987** [20]	Case Report	31 M	AIDS	Sinus cultures, histopathology	Necrotic ulcer on nasal septum	Yes (1)	AmB 1 g total, 3 times per week (unknown)	Cure, but death due to unrelated causes at 8 months
**Loveless et al. 1981** [1]	Case Report	47 M	Chronic granulocytic leukemia, neutropenia	Sinus cultures, histopathology	Lesion on nasal septum	Yes (1)	None	Cure, no follow up
**Shugar et al. 1981** [15]	Case Report	50 F	None	Palate tissue culture, imaging	Necrotic ulcer on hard palate, right nasolabial fold mass, and oronasal fistula	Yes (3)	AmB (91), then multiple AmB courses after more relapses, then KET 400 mg/day (unknown) with AmB 20 mg/day (14) + 5-FU 3 g/day (14), then KET (unknown)	Three relapses, no follow up
**Garau et al. 1977** [16]	Case Report	23 F	None	Sinus cultures, imaging	Necrotic ulcer on hard palate and maxillary osteomyelitis	Yes (1)	AmB 1 mg/kg every other day (50), then AmB after relapse (unknown)	One relapse at 17 months, no follow up

Abbreviations:

5-FU = 5-fluorocytosine, AmB = amphotericin B deoxycholate, L-AmB = liposomal amphotericin B, POS = posaconazole, VOR = voriconazole, ITR = itraconazole, KET = ketoconazole, ISZ = isavuconazole

ALL = Acute Lymphoblastic Leukemia, AML = Acute Myeloid Leukemia, APL = Acute Promyelocytic Leukemia, CML = Chronic Myeloid Leukemia, SCT = stem cell transplant, DM2 = type 2 diabetes mellitus, GVHD = Graft Versus Host Disease, ANC = Absolute Neutrophil Count

NGS = next generation sequencing, PCR = polymerase chain reaction, BAL = bronchoalveolar lavage, NGS = Next Generation Sequencing

*Initially local excision, followed by surgical debridement

Almost all cases underwent at least one surgical debridement in combination with antifungal therapy. Antifungal selection and duration varied: a majority received L-AmB monotherapy while recently published cases received L-AmB monotherapy, combination L-AmB with triazole or L-AmB followed by triazole. The triazoles used were itraconazole (ITR), voriconazole (VOR), or POS. No cases were treated with ISAV. Of two patients who were treated with medical management alone, only one achieved cure. Only one patient underwent surgical resection alone and achieved cure. While most (17/21 cases, 81%) patients who received surgical and medical management achieved cure, some required multiple debridements and L-AmB. The earliest published cases resulted in at least one relapse without long-term follow-up despite surgical resection and L-AmB; however, this may suggest an undiagnosed defect in the host response as no risk factors had been identified [15,16]. No infection-related deaths occurred.

## Discussion

Our case of invasive fungal rhinosinusitis demonstrates the challenges of determining the causative pathogen when microbiologic and histopathologic workup are discordant. First, the importance of investigation with histological and culture-based methods should be emphasized. The patient underwent maxillary biopsy one month prior to her index hospitalization, which showed evidence of acute branching, septate, and filamentous organisms. It is unclear if tissue cultures were sent, but they may have facilitated more timely diagnosis and treatment. Upon subsequent admission to our hospital, empiric antifungal therapy was initiated several days prior to surgical debridement and tissue cultures, which may have reduced the diagnostic yield of cultures. The growth requirements of the offending pathogen, *Alternaria*, were likely not a significant factor as dematiaceous molds can grow on most standard mycological media [21].

In contrast, molecular methods including NGS offer higher sensitivity, with the ability to detect small amounts of genetic material from viable and dead fungal organisms [22]. However, this also increases the detection of contaminants or colonizing organisms. In our case, fungal NGS testing detected *Alternaria* spp., *Candida krusei*, *Botryotrichum* species, *Malassezia restricta*, and *Pichia barkeri*. While molds can share some morphologic characteristics, histological methods can clarify the diagnosis. Between the two molds *Botryotrichum* and *Alternaria*, the globose swellings seen along septate hyphae are more consistent with a dematiaceous mold. Like other molds, *Alternaria* has septate hyphae which branch at 45 °, but differs by its more fragmented appearance, globose structures, and melanized cell well identifiable with a Fontana-Masson stain [23]. Caution should be applied when interpreting the results of a positive Fontana-Masson as non-dematiaceous molds including Cryptococcus, Aspergillus, Fusarium, and Zygomycetes have also been shown to produce melanin [24]. Conversely, *Botryotrichum* is not known to produce melanin nor has it been described as a human pathogen, except in one case of pulmonary *Botryotrichum* in the setting of chronic granulomatous disease [25]. Based on the clinical context and histopathologic findings, *Alternaria* is suspected to be the most likely culprit pathogen, though this could not be microbiologically confirmed.

Normally found in soil, wood, and decaying plant matter, dematiaceous molds including *Alternaria* are significant plant pathogens, but also cause a diverse spectrum of human infections known as phaeohyphomycoses [23]. Clinical manifestations range from superficial infections including onychomycosis and keratitis to brain abscesses and disseminated disease [23]. Immunocompetent patients typically present with allergic fungal sinusitis or allergic bronchopulmonary mycosis, which are commonly due to *Bipolaris* and *Curvularia* [23]. Rarely, dematiaceous molds like *Cladophialophora bantiana* can cause brain abscesses in immunocompetent patients [23]. Among the immunocompromised, *Alternaria* most frequently causes superficial or localized cutaneous infection: in a systematic review of solid organ transplant recipients, 94% of *Alternaria* infections were soft tissue and skin infections [21,23,26]. Cutaneous manifestations vary from ulcerative (typically from inoculation) to painless papulonodules which may represent disseminated infection [21]. Like *Bipolaris* and *Curvularia, Alternaria* is an emerging cause of invasive fungal rhinosinusitis in solid organ transplant (SOT) recipients and patients with hematologic malignancy [21,26,27]. Based on case reports and series, a majority of adult cases with invasive *Alternaria* rhinosinusitis are immunocompromised with prior stem cell transplantation or acute leukemia [[1], [2], [3], [4], [5], [6], [7], [8],^18^]. *Alternaria* can cause chronic invasive rhinosinusitis with more indolent destruction of the paranasal sinuses and adjacent tissues over several months [27].

According to the 2021 global guideline by the European Conference of Medical Mycology, International Society for Human and Animal Mycology, and the American Society for Microbiology, VOR monotherapy or L-AmB monotherapy or combination with triazoles or echinocandins are moderately recommended as first-line for phaeohyphomycosis overall [28]. Alternatively, a combination of POS or VOR with echinocandins are moderately recommended as other first-line agents for disseminated infection [28]. In contrast, ISAV is marginally recommended as second-line therapy due to a lack of clinical data [28]. One published case of invasive *Alternaria* sinusitis was successfully treated with ISAV as salvage therapy [29].

MICs can also guide treatment options, although there are no clinical breakpoints to define MIC cutoffs associated with susceptibility. For *Alternaria* isolates in SOT recipients, Radcliffe et al. showed more favorable MICs for POS (0.125–0.25 μg/mL) and L-AmB (0.04–2.0 μg/mL) than fluconazole (2 to > 64 μg/mL) [26]. VOR MICs were variable (0.125–4 μg/mL) [26]. No information was available for ISAV. Initially, our patient was treated with ISAV due to its more favorable side effect profile, but switched to POS based on more favorable clinical data and ISAV intolerance. After 5 surgical debridements and an extended duration of POS, our patient achieved clinical cure. Antifungal therapy should be continued until clinical, radiographic, and histopathologic clearance of infection.

Healthy patients who develop severe or invasive phaeohyphomycoses should undergo immunologic evaluation to elucidate potential primary immunodeficiency disorders. Our patient appeared healthy, but reported a history of mucosal infections in childhood and refractory pansinusitis in adulthood. To investigate primary T-cell or B-cell deficiencies and phagocytic disorders, immunologic workup initially comprised lymphocyte enumeration, immunoglobulin levels, Mendelian susceptibility to mycobacterial disease panel, neutrophil oxidative function test, and Leukocyte Adhesion Deficiency panel followed by mitogen/antigen proliferation testing and whole exome sequencing. Immunologic workup revealed several primary immunodeficiencies: heterozygous CARD9 gene variant (c.723dupG) on whole exome sequencing, lymphopenia, impaired T-cell function, and panhypogammaglobulinemia, conferring defects in the innate and adaptive immune system. CARD9 or caspase recruitment domain-containing protein 9 is an adaptor protein that mediates signals from pattern recognition receptors Dectin-1, Dectin-2, and macrophage-inducible C-type lectin by stimulating cytokine release in response to fungal pathogens [9]. CARD9 deficiency is autosomal recessive, resulting in protein loss-of-function, leading to impaired neutrophil function and recruitment and killing by monocytes and macrophages [9,23]. To date, this is the only mutation with pathognomonic association with phaeohyphomycosis, but also chronic and recurrent candidiasis and dermatophytosis [23]. Compared to homozygous mutations classically found in autosomal recessive CARD9 deficiency, our patient only had one heterozygous CARD9 variant. This is consistent with prior data suggesting variable phenotypes for CARD9 deficiency or dysfunction depending on gene dosage [30]. Our experience highlights the importance of immunologic investigation to determine other infectious risks and to guide preventive and immunomodulatory strategies.

## Conclusion

Accurate diagnosis of invasive fungal infections relies on a combination of histologic and culture methods. Molecular diagnostics can facilitate identification of rare fungal pathogens but may also introduce diagnostic uncertainty when multiple pathogens are detected. In our case, histologic methods and clinical context were essential to clarifying *Alternaria* as the most likely etiology of invasive fungal rhinosinusitis. This case also contributes to the growing literature on invasive *Alternaria* rhinosinusitis in the setting of combined immunodeficiency and heterozygous CARD9 deficiency, successfully treated with surgery and extended triazole therapy. Refractory sinus infections with progressive destruction of surrounding tissue should elicit a high index of suspicion for occult mold infections, including dematiaceous molds.

## Case reports

This research follows the CARE guidelines and the CARE checklist.

## CRediT authorship contribution statement

**Chelsea Morinishi:** Writing – review & editing, Writing – original draft, Visualization, Methodology, Investigation, Conceptualization. **Omai B. Garner:** Writing – review & editing, Investigation. **Shangxin Yang:** Writing – review & editing, Investigation. **Paul R. Allyn:** Writing – review & editing, Supervision.

## Ethics declaration

Written informed consent to take part in the study and to publish the article has been obtained from all participants or their legal representatives. The privacy rights of participants have been observed.

This study was performed in compliance with relevant laws, regulatory frameworks and guidelines where the research took place. The UCLA Institutional Review Board granted an exemption. The authors provided the following explanation: As this study involves human subjects, this study (#24–000091) was reviewed by the UCLA Institutional Review Board and deemed exempt due to minimal risk.

## Informed consent

Written informed consent was obtained from the patient for publication of this case report and accompanying images. A copy of the written consent is available for review by the Editor-in-Chief of this journal on request.

## Funding

We received no financial support for the research and authorship of this article.

## Declaration of Competing Interest

The authors declare that they have no known competing financial interests or personal relationships that could have appeared to influence the work reported in this paper.

## Data Availability

The data are not publicly available due to privacy and ethical restrictions and the local institution’s data policy. The data will be shared on reasonable request from the corresponding author.
